# 

**Published:** 1895-02

**Authors:** 


### CONTENTS.

### COMMUNICATIONS.

Harmony........ 53
Electrical Fusion of Porcelain........ 69
Some Thoughts on the Teaching of Histology and Anatomy in Dental Colleges........ 78
Separators........ 81
The Teeth of Our School Children; What can be Done to Save Them? 95

### SELECTIONS.

Eat Slowly........ 68
Twelfth International Medical Congress........ 68
New Method of Staining Micro-Organisms in the Blood........ 98
Grief, Emotion and Infect.on........ 99
The American Medical Association........ 100
Medical, Pharmaceutical and Dental Directory........ 100
Notice........ 101

### EDITORIAL.

The Academy of Stomatology........ 101
The Louisiana State Dental Society........ 102
Obituary—William O. Kulp........ 102
In Memorium—Dr. Kulp........ 103

